# Perturbation of maternal PIASy abundance disrupts zygotic genome activation and embryonic development via SUMOylation pathway

**DOI:** 10.1242/bio.048652

**Published:** 2019-10-22

**Authors:** Chika Higuchi, Mari Yamamoto, Seung-Wook Shin, Kei Miyamoto, Kazuya Matsumoto

**Affiliations:** 1 Laboratory of Molecular Developmental Biology, Faculty of Biology-Oriented Science and Technology, Kindai University, Wakayama 649-6493, Japan; 2 Laboratory of Cellular and Developmental Biology, National Institute of Diabetes and Digestive and Kidney Diseases, National Institutes of Health, Bethesda, Maryland 20892, USA

**Keywords:** Maternal-to-zygotic transition, PIASy, SUMOylation, Ubiquitin-proteasome system, Zygotic genome activation

## Abstract

During the maternal-to-zygotic transition (MZT), mRNAs and proteins stored in oocytes are degraded and zygotic genes are activated. We have previously shown that the ubiquitin-proteasome system (UPS)-mediated degradation of maternal proteins plays a role in the onset of zygotic transcription. However, it is still unclear which maternal proteins should be degraded for zygotic genome activation and ensuring subsequent embryonic development. In this study, we screen for these maternal factors that are degraded via the UPS. We thus identified a maternal protein PIASy (protein inhibitor of activated STATy), which is an E3 SUMO ligase. The overexpression of PIASy in fertilized embryos causes developmental arrest at the two-cell stage due to severe abnormal chromosome segregation and impaired zygotic transcription. We find that this developmental role of PIASy is related to its SUMOylation activity. Moreover, PIASy overexpression leads to increased trimethylation of histone H3 lysine 9 (H3K9me3) in two-cell nuclei and enhanced translocation of H3K9me3 methyltransferase to the pronucleus. Hence, PIASy is a maternal factor that is degraded after fertilization and may be important for the proper induction of zygotic genome activation and embryonic development.

## Abstract (precis)

**Summary:** Maternal protein degradation is one developmental event leading to acquiring totipotency. PIASy is identified as a maternal protein that is degraded for accomplishing zygotic transcription and development.

## INTRODUCTION

Oocytes can acquire totipotency through fertilization by sperm. During the maternal-to-zygotic transition (MZT), maternally inherited RNAs/proteins stored in oocytes are gradually replaced with zygotic transcripts and proteins produced from the genome of the fertilized oocytes, referred to as zygotic genome activation (ZGA). In order to understand the process of acquiring totipotency, factors that contribute to ZGA have been investigated. Dynamic chromatin remodeling plays a role in ZGA (Eckersley-Maslin et al., 2018). Furthermore, using the two-cell-like cells that are found in the embryonic stem cell population, DUX family transcription factors (human DUX4 and mouse DUX) have been shown as key inducers of genes activated at ZGA by regulating endogenous retroviruses (ERVs) (De Iaco et al., 2017; Hendrickson et al., 2017), while DUX has only a minor role in ZGA in mouse embryos using *Dux* knockout mice (Chen and Zhang, 2019). Thus, transcriptional activation of the zygotic genome seems regulated by multiple factors, but the mechanisms underlying ZGA have not been fully elucidated.

In oocytes, maternal mRNAs and proteins are abundantly accumulated, and subsequently those are degraded after fertilization. These processes are known to modulate development until the regulation by zygotic product. It is well known that in RNA degradation, 90% of maternal mRNA is degraded until the mid-two-cell stage in mouse embryos (Bettegowda and Smith, 2007). The degradation of maternal protein is also important for the transition. On the other hand, ZGA requires maternal mRNAs and a certain set of transcriptions factors (Jimenez et al., 2015; Lu et al., 2016). These observations again highlight the complex regulation mechanisms of ZGA. Among the mechanistic regulations of ZGA, we especially focus on degradation of maternally stored transcriptional repressors. In *Caenorhabditis elegans* embryos, the degradation of a transcriptional repressor OMA-1/OMA-2 is needed for transcriptional activation of the zygotic genome (Guven-Ozkan et al., 2008). Further, autophagy as well as the ubiquitin-proteasome system (UPS) plays a role in maternal protein clearance. Multiple studies have shown that the proteasome- and ubiquitination-related proteins, including those specifically expressed in oocytes or zygotes, are important for early embryonic development (Roest et al., 2004; Shin et al., 2013; Yang et al., 2017; Yu et al., 2013). Moreover, we have previously demonstrated that UPS-mediated protein degradation in fertilized oocytes is important not only for the onset of ZGA in early mouse embryos, but also for full-term development of mice (Higuchi et al., 2018; Shin et al., 2010). A recent study also found that maternal proteins, Dppa3/Stella/PGC7, are partially cleaved by the UPS in the cytoplasm and the N-terminal fragment is required for intracellular trafficking in cleavage stages of mouse development (Shin et al., 2017). Thus, UPS-dependent proteolysis plays a crucial role in MZT. However, the mechanism by which UPS-mediated maternal protein degradation is involved in the ZGA in early mouse embryos is not fully understood.

In this study, to identify the maternal proteins, which are degraded by UPS during MZT and can contribute to zygotic transcription, we screened for several candidates by examining the effects of their overexpression on mouse pre-implantation development. Using this approach, we identified PIASy (protein inhibitor of activated STAT y, also known as PIAS4), which caused developmental arrest at the two-cell stage in its overexpressed embryos. PIASy has E3 ligase activity for the small ubiquitin-like modifier (SUMO) and also functions as a transcriptional repressor. The overexpression of PIASy led to a failure of chromosome segregation and zygotic transcriptional activation, which was caused by the enhanced SUMO ligase activity. Furthermore, PIASy-overexpressed embryos showed an increased level of histone H3 lysine 9 trimethylation (H3K9me3) and translocation of an H3K9 methyltransferase enzyme, SUV39H1, to pronuclei, which is a potential target for SUMOylation of PIASy to activate zygotic transcription. Thus, these results suggest that the degradation of maternal PIASy is concomitant with ZGA and the excess amount of PIASy disturbs ZGA and demethylation of H3K9me3, which results in developmental arrest.

## RESULTS

### PIASy overexpression in mouse fertilized oocytes causes early developmental arrest

**Fig. 1.**
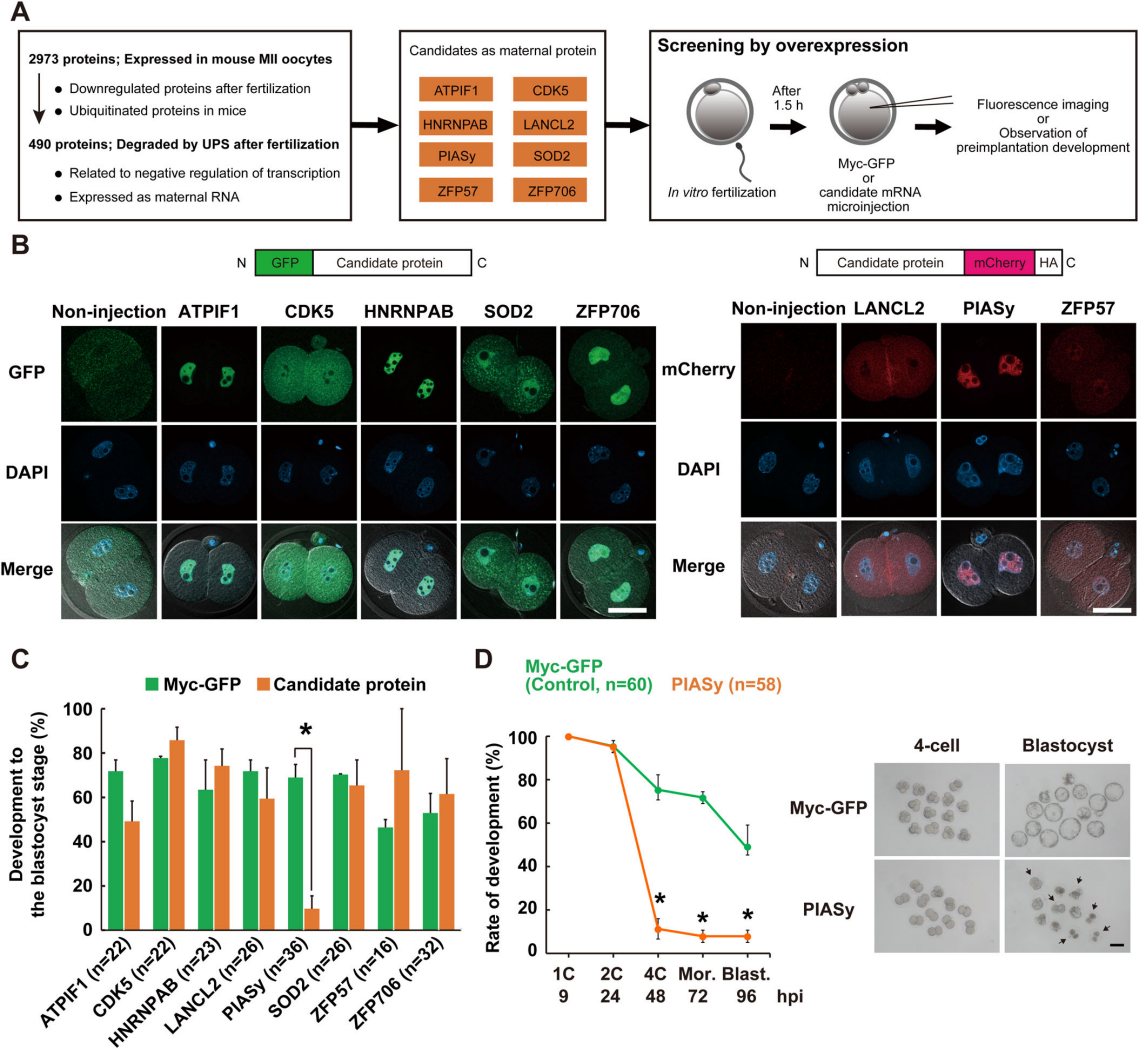
**Effects of PIASy overexpression on mouse pre-implantation development.** (A) Schematic diagram of screening for candidate maternal proteins by bioinformatic approaches and microinjection experiments. (B) Schematic representation of mRNAs encoding candidate proteins that were microinjected into one-cell zygotes just after fertilization (top). Confocal images of GFP-tagged candidate proteins (left panel) and mCherry-tagged candidate proteins (right panel), which were overexpressed in two-cell embryos at 24 hpi. All nuclei were stained with DAPI (blue). Merged images are shown at the bottom panels. Scale bars: 50 µm. (C) Percentages of embryos that developed to the blastocyst stage in eight different candidate genes. *In vitro*-transcribed mRNAs of *Atpif1*, *Cdk5*, *Hnrnpab*, *Lancl2*, *Piasy*, *Sod2*, *Zfp57* and *Zfp706* mRNA were microinjected into the cytoplasm of zygotes. Data are representative of at least two independent experiments. Shown are statistically significant differences between Myc-GFP mRNA and candidate protein-coding mRNA-injected embryos (unpaired two-tailed *t*-test; *P<0.05). Bars represent the standard error of the mean (mean±s.e.m.). Myc-GFP mRNA (green bars), candidate protein-coding mRNA (orange bars). The number of embryos used in each condition is shown in parentheses. Furthermore, the total number of Myc-GFP-injected zygotes for injection control was: ATPIF1, *n*=25; CDK5, *n*=27; HNRNPAB, *n*=25; LANCL2, *n*=25; PIASy, *n*=36; SOD2, *n*=27; ZFP57, *n*=19; ZFP706, *n*=42. (D) Rates of development in PIASy-overexpressed embryos (orange) and Myc-GFP control embryos (green) (left panel). Statistically significant differences between Myc-GFP mRNA- and Piasy mRNA-injected embryos are shown (unpaired two-tailed *t*-test; *P<0.05). Bars represent s.e.m. Representative images of PIASy-overexpressed embryos and Myc-GFP-injected embryos at 48 and 120 hpi (right panel). Arrows indicate arrested embryos at the two-cell stage. Scale bar: 100 µm.

Maternal proteins, which are degraded by the UPS during MZT and can contribute to zygotic transcription and normal development, have not been identified in mice. Firstly, to identify such maternal proteins, we screened for several candidates by examining the effects of their overexpression on mouse pre-implantation development. 2973 candidate proteins were selected from the previously reported proteome datasets in mouse unfertilized oocytes at the metaphase II (MII) stage and fertilized oocytes (Wang et al., 2010a). Proteins for this study were selected based on the following criteria: they were possibly degraded by the UPS after fertilization and were related to transcriptional repression based on the hypothesis in this study: that ZGA occurs by the degradation of the transcriptional repressor (Fig. 1A, see Materials and Methods for details). We finally narrowed down maternal proteins that did meet the criteria (Fig. 1A; Table S1) and decided to test ATPIF1, CDK5, HNRNPAB, LANCL2, PIASy, SOD2, ZFP57 and ZFP706. Expression vectors for green fluorescent protein (GFP: N-terminal)-tagged or monomeric Cherry (mCherry: C-terminal)-tagged full-length candidate proteins were constructed, and subsequently mRNAs were produced by *in vitro* transcription. Fluorescent proteins were tagged either to the N- or C-terminus by avoiding the functional domains. mRNAs corresponding to each candidate gene were injected into the cytoplasm of mouse zygotes 1.5–3 h after fertilization. The expression of eight candidates showed that their GFP or mCherry signals were detected by fluorescence imaging and these synthesized proteins were expressed at the predicted molecular weight by western blotting (Fig. 1B; Fig. S1). We then investigated the effect of overexpression on mouse pre-implantation development. In the seven candidate proteins, no significant difference was observed in the blastocyst rate after overexpression, as compared with myc-tagged GFP (Myc-GFP) mRNA-injected embryos for injection control (Fig. 1C). Meanwhile, the blastocyst rate of PIASy-overexpressed embryos was significantly lower (10%, *n*=36) than that of Myc-GFP injected embryos (69%, *n*=36) (Fig. 1C). Furthermore, only 11% (*n*=58) of embryos developed to the four-cell stage at 48 h post insemination (hpi) and consequently, developmental arrest at the two-cell stage was observed in PIASy-overexpressed embryos (Fig. 1D). Together, we identified PIASy as a candidate maternal factor that is degraded after fertilization for developmental progression.

### PIASy is expressed as a maternal protein and is degraded by the ubiquitin-proteasome system after fertilization

**Fig. 2.**
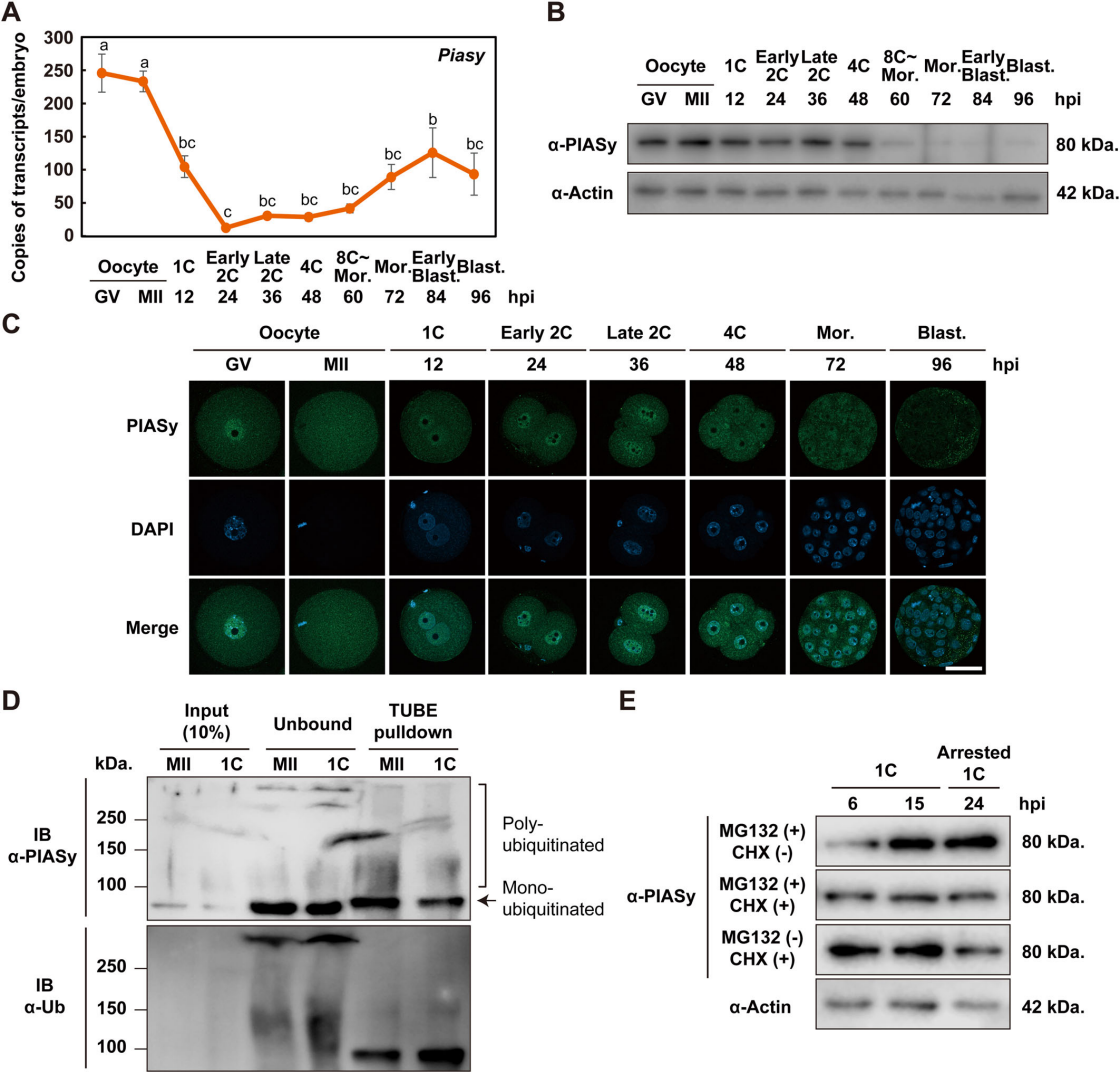
**Maternal PIASy is degraded by UPS after fertilization.** (A,B) Expression profiles of Piasy mRNA (A) and protein (B) in mouse oocytes and early embryos, determined by RT-qPCR using eight oocytes or embryos per stage, and western blot analysis using 30 oocytes or embryos per stage. Data represent mean±s.e.m. and different letters indicate statistical significances (Tukey–Kramer test; *P*<0.05). Actin was used as a loading control. Molecular masses (kDa) are shown on the right. (C) Subcellular localization of PIASy (green) in oocytes at the germinal vesicle (GV) and MII stages and in embryos till the blastocyst stage at 96 hpi (more than ten embryos were examined in each condition). All nuclei were stained by DAPI (blue). Scale bar: 50 µm. (D) Endogenous poly- and mono-ubiquitination of PIASy in mouse MII oocytes and one-cell zygotes. 200 oocytes and zygotes were collected and lysed by lysis buffer. The lysates were subjected to TUBE-pulldown assay to enrich for ubiquitinated proteins, followed by an immunoblot with anti-PIASy (upper panel) or anti-Ub antibody (lower panel). Molecular masses (kDa) are shown on the left. (E) One-cell zygotes at 6 hpi were treated with or without cycloheximide (CHX) in the presence of MG132 and lysed at the indicated time. The lysates were subjected to western blot analysis with anti-PIASy antibody. Total proteins from 30 zygotes were loaded in each lane. Representative western blot analysis of PIASy protein levels and one representative actin loading control are shown. MII, metaphase II oocyte; 1C, one-cell; 2C, two-cell; 4C, four-cell; Mor., morula; Blast., blastocyst.

To investigate the expression profile of endogenous *Piasy*, we analyzed the expression level of its mRNA and protein in mouse oocytes throughout pre-implantation stages by reverse transcription-quantitative polymerase chain reaction (RT-qPCR) and western blot analyses. The mRNA expression level was dramatically decreased after fertilization, while the protein expression level was gradually reduced from the four-cell stage onward (Fig. 2A,B; Fig. S2A). The distinct expression pattern between mRNA and proteins from the morula to blastocyst stage might be due to the differential recognition sites for the primers and antibody, respectively; the PIASy antibody recognizes the C-terminal region, whereas the primers used in RT-qPCR analyses amplify exons 5 and 6 of *Piasy*, which are not at the C-terminus. Moreover, protein expression of PIASy was upregulated during oocyte maturation (Fig. S2A), suggesting that PIASy is encoded by dormant maternal mRNA. The subcellular localization of PIASy was observed in the nuclei of the oocytes at the germinal vesicle (GV) stage and those of pre-implantation embryos at the early developmental stages, and the cytoplasmic PIASy was also detected (Fig. 2C).

To ask whether PIASy protein is degraded by the UPS during MZT, firstly, ubiquitination of PIASy was assessed by TUBE (tandem ubiquitin binding entities)-pulldown assay in MII oocytes and one-cell zygotes. We found that the endogenous PIASy was poly- and mono-ubiquitinated in MII oocytes and zygotes (Fig. 2D). Moreover, to examine the degradation after fertilization, we performed treatment with MG132, an inhibitor of the UPS, or cycloheximide (CHX), an inhibitor of new protein synthesis, in one-cell zygotes from 6 hpi, and then checked the expression at 6, 15, and 24 hpi. In zygotes treated with MG132, the amount of PIASy proteins was increased compared to embryos with CHX in the presence of MG132 (Fig. 2E; Fig. S2B). Collectively, these results show that PIASy is a maternally expressed protein, ubiquitinated and degraded by UPS during MZT.

### Enhanced E3 SUMO ligase activity by PIASy overexpression causes abnormal chromosome segregation

**Fig. 3.**
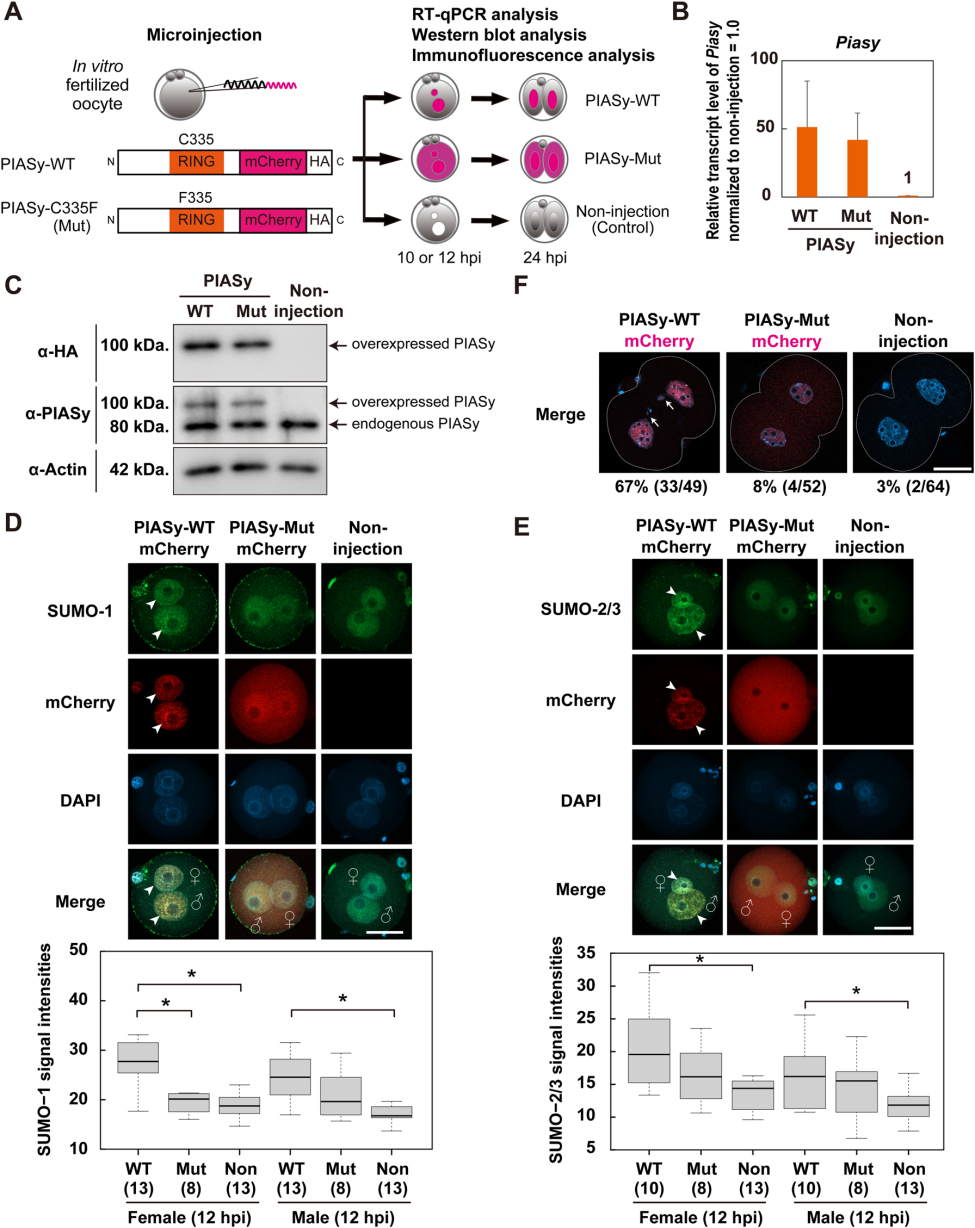
**Enhancement of the E3 SUMO ligase activity of PIASy results in an increased nuclear localization of SUMO.** (A) Schematic diagram of experiments in PIASy-WT or -Mut (C335F) overexpression. The SUMO ligase-inactive point mutant was generated using PCR-based mutagenesis, converting cysteine 335 to a phenylalanine. (B) Overexpression of PIASy-WT or -Mut was confirmed by RT-qPCR using one-cell zygotes. The Piasy mRNA levels of the non-injected one-cell zygotes were defined as 1. Data represent the mean±s.e.m. (C) Western blots of endogenous PIASy and overexpressed mCherry-HA-tagged PIASy in PIASy-WT or -Mut overexpressed zygotes, using anti-HA, anti-PIASy, or anti-Actin antibody. *In vitro*-transcribed mRNAs of Piasy-WT-mCherry-HA or Piasy-Mut-mCherry-HA were microinjected into the cytoplasm of zygotes and the injected zygotes were subjected to western blots at 10 hpi. Actin was used as a loading control. (D,E) Subcellular co-localization of overexpressed PIASy-WT and endogenous SUMO-1 (D) and SUMO-2/3 (E) in one-cell zygotes at 12 hpi. Shown are representative images of zygotes stained with anti-SUMO-1 antibody or anti-SUMO-2/3 antibody immunostaining (green). All nuclei were stained with DAPI (blue). Lower panels show the quantification of SUMO-1 and SUMO-2/3 intensities in pronuclei of examined zygotes (more than eight zygotes were examined). Significant differences were analyzed by Tukey–Kramer tests (*P<0.05). ♀, female pronucleus; ♂, male pronucleus; Scale bars: 50 µm. Arrowheads indicate small dot-like localization of PIASy-WT and SUMO-1 or SUMO-2/3. (F) Overexpression of PIASy-WT shows abnormal chromosome segregation. Shown are representative merged images of mCherry (red) and DAPI (blue) in PIASy-WT (left panel, *n*=49), PIASy-Mut (middle panel, *n*=52), and non-injected two-cell embryos (right panel, *n*=64). Scale bar: 50 µm. Arrows indicate DNA fragments generated during the first cell division. The white dashed line indicates cytoplasmic membrane.

PIASy has E3 ligase activity for the small ubiquitin-like modifier (SUMO) which is one of the post-translational modifications, and has been shown to repress or stabilize the activity of some specific transcription factors and chromatin regulators (Jackson, 2001). This led us to hypothesize that the development of fertilized oocytes would be affected by the PIASy overexpression due to its SUMO ligase activity. To test this, we first constructed the C335F mutant (Mut) of the RING finger domain, which is one of the functional domains for SUMO ligase (Sharrocks, 2006; Yan et al., 2015) (Fig. 3A). Firstly, similar expression levels of mRNA and protein were observed in PIASy-WT and PIASy-Mut-expressed one-cell zygotes (Fig. 3B,C). The amount of exogenously expressed PIASy-WT and PIASy-Mut was lower than that of endogenous PIASy (Fig. 3C; Fig. S3A). Since the overexpressed amount is relatively low, the developmental arrest in PIASy-WT-overexpressed two-cell embryos is unlikely caused simply by the non-physiological amount of overexpressed proteins, but rather by other specific actions of exogenously expressed PIASy-WT. Next, to validate E3 SUMO ligase activity in PIASy-WT and PIASy-Mut, we performed a protein localization assay using anti-SUMO-1 or SUMO-2/3 antibody (since SUMO2 and SUMO3 are highly homologous, this antibody recognizes both the SUMO2 and SUMO3) in PIASy-WT- or PIASy-Mut-overexpressed one-cell zygotes. The signal intensities of the SUMO-1 or SUMO-2/3 in the nucleus of PIASy-WT-overexpressed one-cell zygotes were significantly higher than those of PIASy-Mut or non-injection, although these SUMO proteins showed no changes in the localization among these groups (Fig. 3D,E). Further, in PIASy-WT-overexpressed one-cell zygotes, co-localization of SUMO-1 or SUMO-2/3 with PIASy (merged panel) was observed as small dot-like structures in both male and female pronuclei, whereas SUMO-1 or SUMO-2/3 were not co-localized with PIASy-Mut (Fig. 3D,E, upper panel). Notably, mCherry signals of PIASy-WT and PIASy-Mut exhibited differential cellular localization (Fig. 3D,E). PIASy-WT mainly localized in nuclei, while PIASy-Mut localized in both nuclei and the cytoplasm, in accordance with previous studies on an inactive mutant in the RING finger domain of another PIAS family (Duval et al., 2003). Of note, PIASy-WT-overexpressed two-cell embryos exhibited severe abnormal chromosome segregation (ACS) as is evident from micronuclei and lagging chromosomes (arrows, Fig. 3F; Fig. S3B; 67%, *n*=49), but in PIASy-Mut and non-injection control two-cell embryos had a normal nuclear morphology (Fig. 3F; PIASy-Mut 8.0%, *n*=52; non-injection 3.1%, *n*=64). Thus, these observations indicate the SUMO ligase activity might be one of the causes of developmental arrest in the PIASy-WT-overexpressed embryos. Indeed, PIASy mediates SUMOylation of a chromosome segregation protein, such as Topoisomerase-II, and regulates the decatenation of centromeric DNA (Azuma et al., 2005; Ryu et al., 2010b), suggesting that the SUMOylation activity of PIASy-WT could affect the first mitosis. Based on these results, the loss of the SUMOylation activity of PIASy after fertilization is important for the correct SUMO nuclear localization and proper chromosome segregation during the first mitosis.

### Overexpression of PIASy after fertilization results in abnormal activation of major ZGA genes

**Fig. 4.**
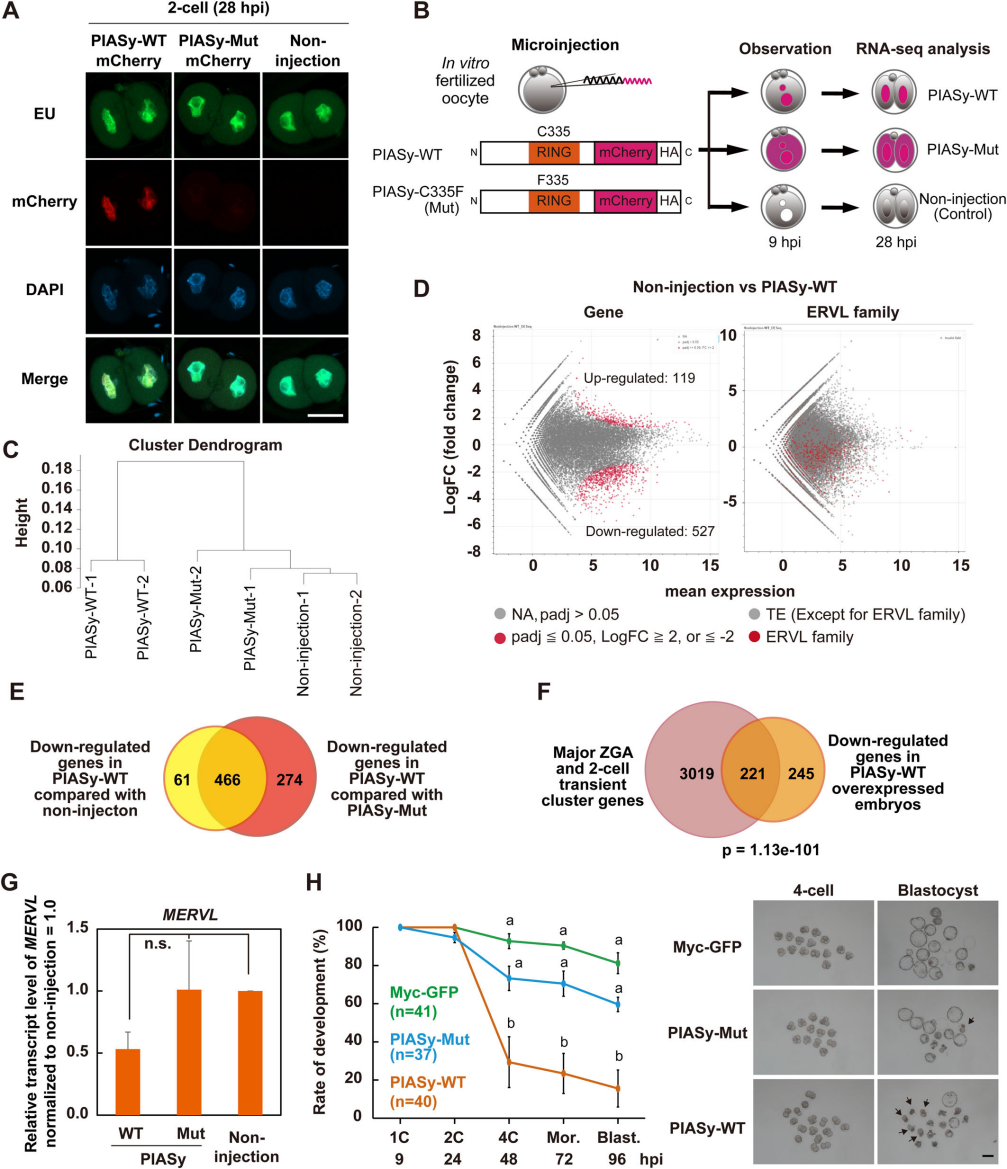
**Effects of overexpressed PIASy after fertilization on zygotic genome activation.** (A) EU incorporation in two-cell embryos at 28 hpi, overexpressed with PIASy-WT or PIASy-Mut. Non-injected embryos were used as a control. Shown are representative images of embryos stained with EU incorporation (green). All nuclei were stained with DAPI (blue). Scale bar: 50 µm. (B) Experimental scheme of RNA-sequencing using PIASy-WT- or -Mut-overexpressed two-cell embryos at 28 hpi. *n*=2. (C) Hierarchical clustering of PIASy-WT-overexpressed, PIASy-Mut-overexpressed, and non-injected embryos based on the global gene expression profile, as revealed by RNA-seq analysis. (D) MA plot displaying differentially expressed genes (left panel) and ERVL family retrotransposons (right panel) in non-injected versus PIASy-WT-overexpressed embryos. Differentially expressed genes with adjusted padj <=0.05 and log FC >=2, or <=−2 are highlighted in red. padj >0.05 are highlighted in gray. ERVL family retrotransposons are highlighted in red, while the other retrotransposons are in gray. (E) Venn diagram shows 466 overlapping downregulated genes after PIASy-WT overexpression, identified by comparing the commonly downregulated genes between PIASy-WT versus non-injection and PIASy-WT versus PIASy-Mut. (F) Venn diagram shows the comparison between the 466 commonly downregulated and 3240 major ZGA and two-cell transient cluster genes extracted from DBTMEE (hypergeometric test; *P*=1.13e-101). (G) The graph of RT-qPCR analyses in PIASy-WT, -Mut, and non-injected control two-cell embryos at 28 hpi. The *MERVL* mRNA levels of the non-injected embryos were defined as 1. n.s., non-significant (Tukey–Kramer analysis). Data represent the mean±s.e.m. (H) Rates of development in PIASy-WT (orange) or -Mut (blue) and Myc-GFP control (green) embryos (more than 37 embryos were tested). Different letters indicate statistical significances (Tukey–Kramer test; *P*<0.05). Bars represent s.e.m. Representative images of PIASy-WT, -Mut embryos and Myc-GFP injected embryos at 48 and 120 hpi (right panel). Arrows indicate arrested embryos at the two-cell stage. Scale bar: 100 µm.

To examine the effect of overexpressing PIASy on ZGA, global transcription activities in two-cell embryos at 28 hpi, at which ZGA had occurred, were measured by 5-ethynyluridine (EU) incorporation. EU levels in the PIASy-WT, and PIASy-Mut-overexpressed two-cell embryos were not markedly different from those of the control group (Fig. 4A; Fig. S4A). These results indicate that the overexpression of PIASy did not affect the global transcription level. Then, we performed RNA-sequencing (RNA-seq) analysis of PIASy-WT, and -Mut overexpressed, and non-injected two-cell embryos at 28 hpi to ask if a specific set of genes were misregulated (Fig. 4B). Hierarchical clustering based on their gene expression profile showed that PIASy-WT samples were clearly separated from the non-injection control and PIASy-Mut samples (Fig. 4C). In addition, the heat map showed that gene expression was highly correlated between the non-injection control and PIASy-Mut embryos, whereas the transcriptome of PIASy-WT-overexpressed embryos was different from that of others (Fig. S4B). Furthermore, PIASy-WT overexpression induced downregulation of 527 and 740 genes (log_2_ fold change <=−2, padj <=0.05) as compared with the non-injection control and PIASy-Mut, respectively (Fig. 4D; Fig. S4C, left panel). We identified 466 downregulated genes in PIASy-WT-overexpressed embryos, which were common to both non-injection and PIASy-Mut (Fig. 4E). We then compared the 466 gene list, a major ZGA gene list and a two-cell transient gene list from a database of transcriptome in mouse early embryos (DBTMEE). Hypergeometric tests showed that the downregulated genes were significantly enriched in the major ZGA gene list and two-cell transient gene list (*P*=1.13e-101), indicating that the overexpression of PIASy-WT leads to a failure of activating at least part of major ZGA genes (Fig. 4F). Mouse endogenous retrovirus type L (MERVL) is one class of retrotransposable elements (TEs) and is abundantly expressed in mouse two-cell stage embryos, and it drives the expression of many transcripts specific to ZGA and totipotency (Kigami et al., 2003; Macfarlan et al., 2012). Thus, we investigated whether PIASy-WT overexpression affected the expression of the MERVL family. Based on our RNA-seq results, the expression of the MERVL family was lower in PIASy-WT than in the non-injection or PIASy-Mut, but the other class of TEs had no marked difference between PIASy-WT and non-injection or PIASy-Mut (Fig. 4D; Fig. S4C, right panel). Furthermore, to validate RNA-seq results of the MERVL family, RT-qPCR was conducted in PIASy-WT-overexpressed two-cell embryos. As a result, the expression level of MERVL in PIASy-WT showed lower than that of non-injection and PIASy-Mut by 1.9-fold (Fig. 4G; Fig. S4D). It should be noted, however, that the activation of *Zscan4* cluster genes, which are also cleavage stage-specifically expressed endogenous genes, was not affected by the PIASy-WT overexpression (Fig. S4E). These results suggest that the excess amount of PIASy impairs the induction of early embryonic transcripts.

Next, the upregulated genes after PIASy-WT overexpression were categorized based on the cluster genes in DBTMEE (Table S2). Interestingly, most of the upregulated genes were categorized as minor ZGA genes and one-cell transient genes. We also observed that the *Dux* expression level was increased in PIASy-WT-overexpressed embryos (Fig. S4D). These results imply that minor ZGA genes including *Dux* are continuously expressed in late two-cell embryos after PIASy-WT overexpression. Improper completion of minor ZGA may result in the failure of activating major ZGA genes, as observed in our PIASy-WT-overexpressed embryos.

### Impaired ZGA and embryonic development are attributed to the enhancement of the E3 SUMO ligase activity of PIASy

The overexpression of PIASy-WT was accompanied with the enhanced E3 SUMO ligase activity (Fig. 3D,E). We then asked whether the PIASy-WT overexpression induced the misexpression of SUMO-conjugate or SUMO protease genes, such as the *Pias*, *Sumo*, *Sae*, *Ube* and *Senp* family. However, we did not detect effects of PIASy-WT overexpression on the expression of most of the tested factors, except for *Pias3*, *Senp2* and *Senp8*, which are classified as late zygotic or maternally expressed genes, based on the RNA-seq analysis (Fig. S4F). Thus, SUMOylation genes or SUMO protease genes other than PIASy unlikely induced the phenotype related to the PIASy-WT overexpression. Lastly, we tested whether the SUMO ligase activity of PIASy affected the pre-implantation development. The developmental rate of PIASy-WT-overexpressed embryos was significantly lower than that of the control embryos in the same manner as in Fig. 1D (Fig. 4H, 14%, *n*=40). Conversely, no significant difference in the developmental rate was found between PIASy-Mut (63%, *n*=37) and Myc-GFP-injected control (83%, *n*=41), and PIASy-Mut showed normal blastocyst morphology (Fig. 4H). Thus, these results indicate that the overexpression of PIASy, which leads to inhibition of embryonic development, is attributed to its E3 SUMO ligase activity, and the activity can negatively regulate zygotic transcription.

### Overexpression of PIASy leads to the translocation of endogenous SUV39H1 to pronuclei and affects the heterochromatin state

**Fig. 5.**
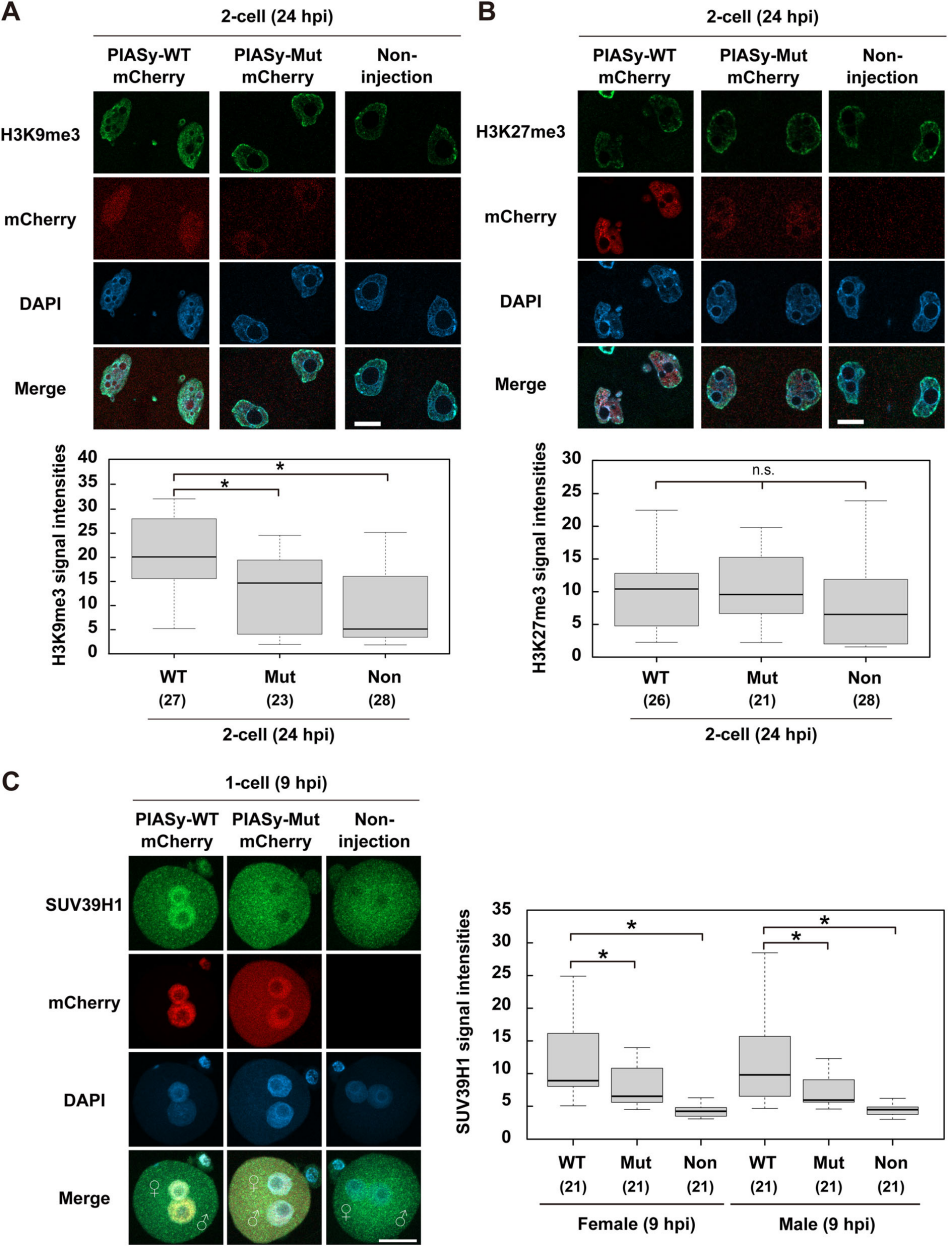
**Overexpression of PIASy affects a heterochromatin state, and endogenous SUV39H1 is translocated to pronuclei of PIASy-overexpressed zygotes.** (A,B) Immunofluorescence analysis of endogenous H3K9me3 (A) and H3K27me3 (B) in two-cell embryos at 24 hpi overexpressed with or without PIASy-WT/PIASy-Mut. Shown are representative images of embryos stained with anti-H3K9me3 or anti-H3K27me3 antibody (green). All nuclei were stained with DAPI (blue). Scale bars: 20 µm. Lower panels show the quantification of H3K9me3 and H3K27me3 intensities in nuclei of examined embryos at 24 hpi (more than 21 embryos were tested). Significant differences were analyzed with Tukey–Kramer tests (*P<0.05). n.s., non-significant. (C) Subcellular localization of endogenous SUV39H1 in one-cell zygotes at 9 hpi, overexpressed with PIASy-WT or PIASy-Mut. Shown are representative images of zygotes stained with anti-SUV39H1 antibody (green). All nuclei were stained with DAPI (blue). ♀, female pronucleus; ♂, male pronucleus. Scale bar: 50 µm. Right panel shows the quantification of SUV39H1 intensities in pronuclei of examined zygotes at 9 hpi (21 zygotes were tested). Significant differences were analyzed with Tukey–Kramer tests (*P<0.05).

PIASy is known to associate with heterochromatin (Bischof et al., 2006). We therefore examined the constitutive heterochromatin mark H3K9me3 in PIASy-WT-overexpressed two-cell embryos by immunofluorescence analysis. PIASy-WT-overexpressed two-cell embryos showed a significant increase in intensities of H3K9me3 staining in two-cell nuclei in comparison to the PIASy-Mut and non-injection control (Fig. 5A). We also observed a similar trend in the signal intensity of female pronuclei in PIASy-WT-overexpressed one-cell zygotes, while the signal in male pronuclei, which is naturally low, was unaltered (Fig. S5A). To ascertain the facultative heterochromatin marker H3K27me3, we then analyzed the intensity level of H3K27me3. There was no significant difference in the signal intensity of H3K27me3 among these groups, indicating that PIASy-WT did not affect H3K27me3-related factors (Fig. 5B; Fig. S5B). Overall, these results indicate that PIASy-WT overexpression can cause an increase in H3K9me3-mediated heterochromatin formation, suggesting that SUMOylation by PIASy-WT is involved in H3K9 trimethylation-associated factors.

We finally asked which protein is targeted by PIASy in early mouse embryos. In mammalian cells, H3K9 methyltransferase enzymes including SUV39H1 (suppressor of variegation 3-9 homolog 1), SUV39H2, and SETDB1 catalyze H3K9 trimethylation, whereas histone lysine demethylases, 4A (KDM4A) and KDM4D, promote demethylation of H3K9me3 (Dimitrova et al., 2015; Zhang et al., 2015). These ‘writers’ and ‘erasers’ for H3K9me3 are highly expressed in oocytes and two-cell embryos (Wang et al., 2018), suggesting that these factors may play a role in H3K9me3 reprogramming of early mouse embryos. To determine whether the overexpression of PIASy-WT affects such factors, we examined the localization of endogenous SUV39H1, a H3K9 methyltransferase in PIASy-WT-overexpressed embryos by immunofluorescence analysis. In PIASy-WT-overexpressed one-cell zygotes, the localization of SUV39H1 was significantly increased in both male and female pronuclei, as compared with PIASy-Mut and non-injection control (Fig. 5C). The same results were obtained in two-cell embryos (Fig. S5C). Additionally, endogenous SUV39H1 was largely co-localized with PIAS-WT in the nucleus (Fig. 5C). These results indicate that the overexpression of PIASy-WT provokes the nuclear accumulation of SUV39H1 via its SUMOylation activity, which in turn results in an increased level of H3K9me3.

## DISCUSSION

**Fig. 6.**
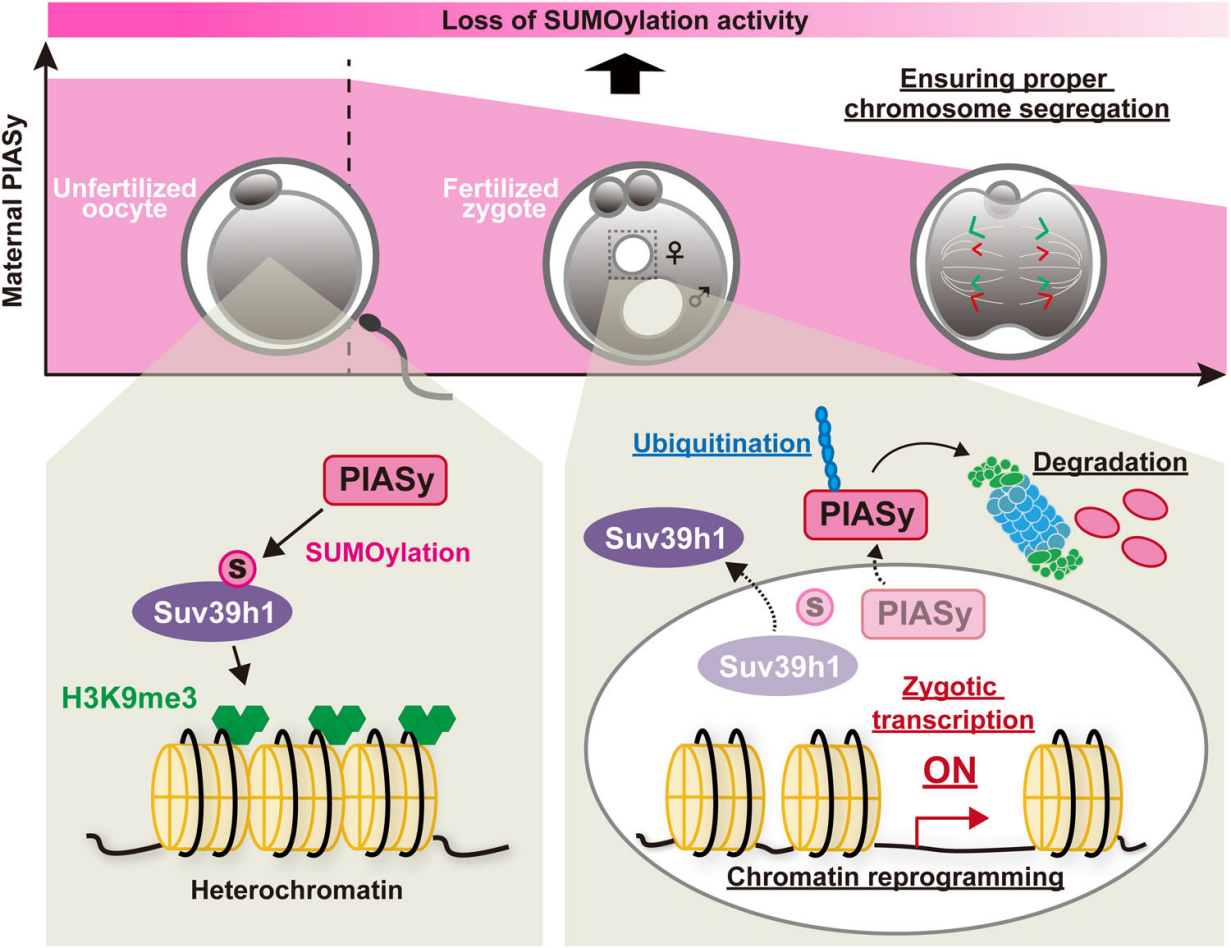
**A model of maternal PIASy-mediated regulation during maternal-to-zygotic transition.** In unfertilized mouse oocytes, maternally expressed PIASy possibly stabilizes a histone methyltransferase for H3K9me3 via the SUMOylation activity (yet it remains unclear). After fertilization, PIASy is degraded by the UPS, marked by ubiquitination. At the same time, elimination or inhibition of the SUMO ligase activity of PIASy may lead to reprogramming of H3K9me3-dependent heterochromatin, zygotic genome activation and proper chromosome segregation. The underlined events in the diagram are investigated in this study.

Various kinds of maternal proteins are degraded during MZT for progressing embryonic development. However, it has been unclear which maternal proteins need to be degraded for accomplishing MZT, and yet the mechanisms by which the degradation of maternal proteins drives MZT are poorly defined. Using the overexpression screening approach, here we show that the excess amount of PIASy impairs mouse pre-implantation development. PIASy is a maternal protein that is accumulated during oocyte maturation and subsequently degraded during pre-implantation development. We propose that the decay of PIASy via UPS is important for modulating the SUMOylation balance after fertilization, and the misregulation of this process may trigger errors in chromosome segregation, zygotic transcription, and chromatin reprogramming during MZT (Fig. 6). In contrast to PIASy, the other seven examined candidate proteins have little effect on early development. A large-scale proteomic analysis indicated that the expression and the destruction of maternal proteins are tightly controlled in a time-dependent manner (Gao et al., 2017). In addition, several maternal proteins are rapidly degraded after ZGA (Bebbere et al., 2016). It is therefore possible that the other tested proteins also need to be degraded for progressing development, but the phenotype is not as severe as PIASy overexpression. The variable efficiency in protein degradation could also affect the phenotype.

Our results document that maternal PIASy was expressed at least from the oocyte to eight-cell stage (Fig. 2). However, the excess accumulation of PIASy just after fertilization led to arrest of embryos at two-cell stage (Fig. 1D). These results raise the question of why the developmental arrest occurs at the two-cell stage in the overexpressed embryos although there remains endogenous maternal PIASy in two-cell embryos (Fig. 2B). We found that overexpressed PIASy-WT mainly localized in nuclei, while PIASy-Mut lacking the functional SUMO ligase activity showed both nuclear and cytoplasmic signals (Fig. 3D,E). Endogenous PIASy localized in both nuclei and the cytoplasm in early mouse embryos (Fig. 2C). Thus, these results suggest that the functional or non-functional state of PIASy exists in early mouse embryos. One of the causes of the developmental arrest at the two-cell stage may be due to the overexpression of functional PIASy, which is evident from the predominant nuclear localization pattern in PIASy-WT-expressed embryos. Ubiquitination of a target protein leads to the altered protein function or fates (Sadowski et al., 2012), and the substrates lead to degradation by 26S proteasome following mono-/poly-ubiquitination (Braten et al., 2016; Saeki, 2017). Most proteins destined for degradation are translocated into the cytoplasm (Wu et al., 2017). Indeed, maternal PIASy was mono- and poly-ubiquitinated after fertilization (Fig. 2D) and cytoplasmic PIASy was observed (Fig. 2C). Thus, these observations may explain that ubiquitination and degradation during MZT inhibit the function (i.e. E3 SUMO ligase activity) of endogenous maternal PIASy after fertilization without disturbing normal embryonic development. E3 ubiquitin ligases for ubiquitination and degradation may have a role in the above process. In somatic cells, two E3 ubiquitin ligases e.g. TRIM32 (tripartite motif containing 32) (Albor et al., 2006) and FIEL1 (fibrosis-inducing E3 ligase 1) (Lear et al., 2016) have been reported to serve as E3 ubiquitin ligases for PIASy. However, the E3 ubiquitin ligase activity towards PIASy has not been identified in oocytes and in pre-implantation embryos. It seems important to investigate whether E3 ubiquitin ligases could be an upstream regulator of maternal PIASy to understand the dynamics of endogenous PIASy as well as embryonic development.

The SUMO ligase activity of PIASy profoundly affects early embryonic development (Fig. 4H). SUMOylation is a post-translational modification and it participates in cellular processes such as transcription, nuclear transport, chromatin remodeling, DNA repair and mitosis (Flotho and Melchior, 2013). Like the ubiquitination pathways, SUMO-activating enzyme subunit SAE, conjugating enzyme UBC9, and E3 SUMO ligase cooperatively work for a conjugation of SUMO to target proteins. Further, for reversible SUMOylation, SUMO protease SENP cleaves between SUMO and the target protein (Flotho and Melchior, 2013). SUMO, UBC9 and SENP are known to be expressed in mouse oocytes till pre-implantation stage embryos, and function in oocyte maturation and early embryonic development (Ihara et al., 2008; Wang et al., 2010b). Here, we reveal that SUMO proteins are localized in the female and male pronuclei of one-cell zygotes. In the PIASy-WT-overexpressed zygotes, an increase in SUMO abundance and distinct distribution within the nucleus was concurrent with the PIASy-WT pronuclear localization, compared with those of endogenous PIASy (Figs 2C and 3D,E). Such distinct distribution as small dot-like structures of SUMO-1 or SUMO-2/3 with PIASy-WT can be related to the protein stability, controlled by SUMOylation. In fact, similar observations have been reported in the case of Pax8, which is a target protein of PIASy for regulating its stability (Cristofaro et al., 2009). It has also been shown that the fine-tuning of SUMOylation balance is essential for early development (Yamaguchi et al., 2005). Thus, the manipulation of PIASy expression after fertilization showed a significant effect on developmental events, which could be due to the altered balance of SUMO conjugation-deconjugation during MZT.

We also observed that the overexpression of PIASy led to severe abnormal chromosome segregation as analyzed by confocal images of two-cell stage embryos (Fig. 3F). It is known that PIAS proteins and SUMO-related factors are involved in the mitosis, and that PIASy has its capacity to bind to mitotic chromosomes and recruit other SUMO related-factors onto chromatin. Using *Xenopus* egg extracts, it was shown that PIASy regulates the mitotic chromosomes through SUMOylation of Topoisomerase-II and Poly(ADP-ribose) Polymerase 1, which are required for chromosome assembly and segregation for the decatenation of centromeric DNA (Azuma et al., 2005; Ryu et al., 2010a). Besides, the PIASy depletion study shows that misaligned chromosomes increase at the metaphase plate (Agostinho et al., 2008). Maternal SENP7, which is one of the SUMO proteases, is also important in mouse meiosis and pre-implantation development (Huang et al., 2017). Further, PIAS family proteins including PIASy localize at a kinetochore-like pattern at metaphase II in mouse oocytes (Ding et al., 2018). Thus, we assume that the misaligned chromosomes by an excess amount of PIASy result in abnormal chromosome segregation during the first mitosis, which subsequently causes developmental arrest.

We previously demonstrated that degradation of maternal proteins via UPS is involved in the onset of zygotic genome activation (Higuchi et al., 2018; Shin et al., 2010). The overexpression of PIASy did not affect the novel RNA synthesis in a global level. However, RNA-seq analyses revealed that overexpression of PIASy impaired proper activation of zygotic transcripts, especially major ZGA genes and MERVL family transcripts (Fig. 4). Furthermore, many of the upregulated genes were categorized as minor ZGA genes in PIASy-overexpressed two-cell embryos (Table S2). Therefore, minor ZGA still continues in PIASy-overexpressed two-cell embryos and these embryos fail to proceed to major ZGA. This type of misexpression of minor ZGA genes is similar to a previous report, in which maternal LSD1/KDM1A, a histone demethylase enzyme for H3K4me3 and H3K9me3, was depleted (Ancelin et al., 2016; Wasson et al., 2016). In good agreement with our study, maternal depletion of LSD1/KDM1A results in increased H3K9me3 in zygotes and abnormal gene expression in two-cell embryos, such as upregulated minor ZGA genes and downregulated major ZGA genes. It is also known that the activation of minor ZGA genes at the two-cell stage inhibits the induction of major ZGA (Abe et al., 2018). Altogether, it is plausible that the degradation of maternal PIASy controls the repression for minor ZGA and the subsequent activation for major ZGA through balancing its SUMOylation activity, therefore contributing to MZT by mediating a switch from the maternal to zygotic transcriptional programs.

Heterochromatin structures are reprogrammed during early embryonic development to establish totipotency. Our results revealed that the overexpression of PIASy causes an increase in the constitutive heterochromatin marker H3K9me3 in female pronuclei and two-cell nuclei, and relocalization of endogenous SUV39H1 into pronuclei (Fig. 5; Fig. S5). H3K9me3 should be re-established after fertilization (Wang et al., 2018). However, the abnormally high amount of SUV39H1, caused by overexpression of PIASy, may prevent this rebuilding process of H3K9me3-dependent heterochromatin. The re-establishment of genome structures is important for the regulation of gene expression. The failures in erasing maternal H3K9me3 in PIASy-overexpressed embryos likely result in abnormal genome structures and gene expression. This may explain the continued expression of minor ZGA genes and the unsuccessful progression to the next stage. Furthermore, facultative heterochromatin H3K27me3 was not altered in the PIASy-overexpressed two-cell embryos (Fig. 5B), suggesting that PIASy may be targeted to the specific histone modification factors. Moreover, SUV39H1 in itself promotes SUMOylation of heterochromatin protein 1α, a ‘reader’ of H3K9me3, for organizing heterochromatin formation (Maison et al., 2016). Together, SUMOylation may act as one of the modulators of chromatin reprogramming during MZT.

Our presented data support the idea that the degradation of maternal PIASy after fertilization is needed for early embryonic development. Previously described knockout mouse models of *Piasy* show no apparent developmental phenotype (Roth et al., 2004; Wong et al., 2004). This might be because PIAS1, which is another PIAS family protein, has a compensatory role and overlapping functions (Tahk et al., 2007). Thus, PIASy itself is not required for full embryogenesis, but its timely degradation may be needed for accomplishing the zygotic gene expression program and embryonic development by assisting SUMOylation pathway. Further, we speculate that the UPS-mediated maternal protein degradation is highly orchestrated to progress embryonic development, which is accompanied by balancing SUMOylation. In summary, we find a mechanistic link that connects maternal protein abundance, chromatin remodeling and zygotic transcription during MZT. Furthermore, our findings help to understand the transition of maternal or highly differentiated state to the totipotent state. Here, we observed one aspect of the particular maternal protein. Various maternal proteins support the progression of embryonic development, hence further studies are needed to fully elucidate the underlying mechanism.

## MATERIALS AND METHODS

### Animals

For the collection of oocytes and *in vitro* fertilization, all mice (*Mus musculus*, ICR strain) were purchased from Kiwa Laboratory Animals at >8 weeks of age and maintained in light-controlled, air-conditioned rooms. This study was carried out in strict accordance with the recommendations in the Guidelines of Kindai University for the Care and Use of Laboratory Animals. The protocol was approved by the Committee on the Ethics of Animal Experiments of Kindai University (Permit Number: KABT-26-002). All efforts were made to minimize the number of mice used in the study; all mice were killed by cervical dislocation, ensuring minimum suffering.

### Collection of oocytes, *in vitro* fertilization and embryo culture

For *in vitro* fertilization, spermatozoa were collected from the cauda epididymis of male mice (>8 weeks of age). The sperm suspension was incubated in human tubal fluid (HTF) medium for 1.5 h to allow for capacitation at 37°C under 5% CO_2_. Oocytes were collected from the excised oviducts of female mice (>8 weeks of age) that had been super-ovulated with pregnant mare serum gonadotropin (PMSG; Serotropin, Teikoku Zoki) followed by human chorionic gonadotropin (hCG; Puberogen, Sankyo) 48 h later. Cumulus-oocyte complexes were recovered into pre-equilibrated HTF medium. The sperm suspension was added to the oocyte cultures, and morphologically normal fertilized oocytes were collected 1.5 h after insemination. The fertilized embryos were cultured in potassium simplex optimized medium (KSOM) at 37°C under 5% CO_2_.

### Selection of candidate proteins

Candidate proteins for overexpression screening were selected from the previously published proteome dataset in mouse unfertilized oocytes at the MII stage and fertilized oocytes (Wang et al., 2010a) and narrowed down using the following databases. Mouse ubiquitinated proteins were selected from mammalian post-translational modifications resource (PhosphoSitePlus; https://www.phosphosite.org/homeAction.action). To select proteins' function as a transcriptional repressor, they were selected by Gene Ontology (GO) terms as ‘negative regulation of transcription’ using Database for Annotation, Visualization and Integrated Discovery (DAVID; https://david.ncifcrf.gov). For selection of maternally inherited proteins, expressed as maternal RNA using DBTMEE (Database of Transcriptome in Mouse Early Embryos; http://dbtmee.hgc.jp/). Selected proteins are listed in Table S1.

### Plasmid construction, *in vitro* transcription, and microinjection of synthetic mRNA

To investigate the effects of overexpressing selected maternal proteins on the development of early embryos, each mouse full-length cDNA was fused with GFP or mCherry at the N-terminal or C-terminal, respectively, and cloned into a pCS2 vector (Miyamoto et al., 2011). Each RNA amplification was performed using mMESSAGE mMACHINE SP6 Transcription Kit (Thermo Fisher Scientific) from the pCS2 vectors. Subsequently the RNA samples were polyadenylated using Poly (A) Tailing Kit (Thermo Fisher Scientific). To investigate the effect of overexpression in oocytes immediately after fertilization, each RNA (100 ng/µl) was injected into the cytoplasm of mouse fertilized oocytes from 1.5  hpi to 3 hpi, which were confirmed by extrusion of second polar body, in Chatot-Ziomek-Bavister (CZB)-Hepes medium. PIASy-C335F mutation performed by PCR mutagenesis was using the primers (Table S3). The injected zygotes showing GFP or mCherry signals at 9 hpi after microinjection were selected and then cultured to examine the effect of overexpression on subsequent embryonic development to the blastocyst stage. In these experiments, Myc-GFP mRNA (100 ng/µl) was used as a control RNA.

### Inhibitor treatment

To inhibit the activity of proteasomes in one-cell zygotes, they were cultured in KSOM medium containing 5 µM MG132 (Sigma-Aldrich; C2211). For inhibition of protein synthesis, zygotes were treated with 1 µg/ml cycloheximide (Sigma-Aldrich; C7698). Zygotes were cultured in KSOM medium in the presence of MG132 and CHX, or MG132 and 0.1% DMSO, or CHX and 0.1% DMSO from 6 hpi.

### Western blot and densitometric quantification analyses

Briefly, 15 or 30 embryos at each embryonic stage were subjected to sodium dodecyl sulfate (SDS) polyacrylamide gel electrophoresis for the detection of PIASy or ubiquitinated proteins. The protein extracts were resolved in 7.5 or 10% running gel and electrophoretically transferred to polyvinylidene difluoride (PVDF) membranes (GE Healthcare). The membranes were incubated in Block Ace (Dainippon Sumitomo Pharma) at room temperature (RT) for 1 h. They were washed with phosphate-buffered saline containing 0.2% Tween 20 (PBST), and incubated at 4°C overnight with anti-PIASy antibody (Sigma-Aldrich; SAB4502145), anti-HA antibody (Sigma-Aldrich; H9658), anti-GFP antibody (Novus Biologicals; NB600-597), or anti-Actin antibody (Sigma-Aldrich; A5441) as a loading control. The membranes were washed in PBST, incubated with donkey anti-rabbit IgG horseradish peroxidase (HRP) conjugate (Millipore; AP182P) for anti-PIASy or donkey anti-mouse IgG HRP conjugate (Millipore; AP192P) for anti-HA, anti-GFP, and anti-Actin at RT for 1 h, washed again with PBST, and developed using ECL Prime Western Blotting detection reagent (GE Healthcare). At least three independent experiments were performed for each group. The membranes were imaged with an Odyssey Fc imaging system (LI-COR Biosciences). Antibodies used in this study and dilution factors are listed in Table S4. Densitometric quantification analysis of the immunoblot bands was performed using an Image Studio Lite version 5.0.21 (LI-COR Biosciences).

### TUBE pulldown assay

We collected 200 each of MII oocytes and one-cell zygotes and lysed (on ice) in lysis buffer (100 mM Tris, pH 8.0, 0.15 M NaCl, 5 mM EDTA, 1% NP-40, 0.5% Triton X-100) supplemented with a protease inhibitor cocktail (Roche), PhosSTOP (Roche), 200 µM PMSF, 2 mM 1, 10-phenanthroline, 5 mM N-Ethylmaleimide, 50 µM PR-619 (LifeSensors), and 5 µM anti-M1 TUBE (Tandem Ubiquitin Binding Entities, LifeSensors; UM606). The lysate was diluted fivefold by adding reaction buffer (the above lysis buffer excludes NP-40 and Triton X-100). After 2 h incubation on ice, the supernatant was incubated with anti-FLAG M2 affinity Gel (Sigma-Aldrich; A2220) at 4°C overnight with rotation. The beads were washed with ice-cold wash buffer (100 mM Tris, pH 8.0, 0.15 M NaCl, 5 mM EDTA, 0.05% NP-40) six times and eluted into SDS-sample buffer by boiling for 5 min. For detection of PIASy and poly- and mono-ubiquitinated protein, anti-PIASy and anti-ubiquitin (FK2) antibody (Enzo Life Sciences; BML-PW8810) were used (Table S4).

### Immunofluorescence analysis and microscopy

Subcellular localizations of PIASy, SUMO-1, SUMO-2/3, H3K9me3, H3K27me3, and SUV39H1 were determined by immunofluorescence analysis of early embryos. Embryos were fixed in 4% paraformaldehyde (Nacalai Tesque) in PBS for 15 min at RT, and the fixed samples were then incubated in PBS containing 0.5% Triton X-100 (Nacalai Tesque) at RT for 20 min. For testing PIASy, SUMO-1, SUMO-2/3, and SUV39H1, fixed samples were treated with acid Tyrode's solution (Sigma-Aldrich; T1788) to remove the zona pellucida before permeabilization. They were then incubated with anti-PIASy (Sigma-Aldrich; SAB4502145), monoclonal antibodies SUMO-1 (21C7) (Thermo Fisher Scientific; 33-2400), SUMO-2/3 (8A2) (Abcam; ab81371), anti-H3K9me3 (Abcam; ab8898), anti-H3K27me3 (Active Motif; 39157), and anti-SUV39H1 (Cell Signaling Technology; 8729) in PBS containing 30 mg/ml bovine serum albumin at 4°C overnight. Thereafter, the embryos were incubated with Alexa Fluor 488-labeled goat anti-mouse IgG antibody (Invitrogen; A-11001) for anti-SUMO-1 and SUMO-2/3, and with Alexa Fluor 488-labeled goat anti-rabbit IgG antibody (Invitrogen; A-11008) for anti-PIASy, anti-H3K9me3, anti-H3K27me3, and anti-SUV39H1 antibody, all at RT for 1 h. Specimens were mounted on glass slides in VECTASHIELD mounting medium (Vector Laboratories) containing 3 µg/ml 4′, 6-diamidino-2-phenylindole (DAPI) (Invitrogen; D1306). Finally, the slides were imaged using an LSM800 confocal laser scanning microscope (Zeiss) equipped with an oil objective lens (40×objective). All experiments were performed at least in triplicates. Fluorescence intensities were quantified using ImageJ software (National Institutes of Health) after background subtraction. Antibodies used in this study and dilution factors are listed in Table S4.

### Global transcription assay by EU incorporation

The procedures were performed using a commercial kit (Click-iT RNA Alexa Fluor 488 Imaging Kit, Life Technologies; C10329) as described by the manufacturer. In brief, following microinjection of synthetic mRNA, two-cell embryos were cultured in the presence of 2 mM 5-ethynyl uridine (EU) at 37°C from 26 hpi for 2 h. Embryos were fixed in 4% paraformaldehyde and permeabilized with 0.5% Triton X-100 in PBS at RT for 20 min. Click-iT reaction cocktail was added for incubation at RT for 30 min. Specimens were mounted on glass slides in VECTASHIELD mounting medium containing 3 µg/ml DAPI. At least three independent experiments were performed for each group.

### RT-qPCR analyses

Total RNA was extracted from eight oocytes and embryos from each stage, using PicoPure RNA Isolation Kit (Life Technologies; KIT0204) according to the manufacturer's protocol. cDNA was synthesized from total RNA using Superscript III RT First-Strand Synthesis system (Life Technologies; 18080051), and prepared cDNA samples were amplified and analyzed by RT-qPCR. Amplified products were run in a 7300 ABI Prism Sequence Detector (Applied Biosystems). For absolute quantification of *Piasy* transcripts, plasmid containing *Piasy* cDNA was used as the standard. The primers used are described in Table S3. At least three independent experiments were performed for each group.

### Library preparation for RNA-seq analysis

Five two-cell embryos at 28 hpi after the removal of zona pellucida were subjected to RNA purification and cDNA amplification using SMART-Seq v4 Ultra Low Input RNA Kit for Sequencing (Takara Bio) according to the manufacturer's instructions. At the cell lysis step, 1 µl of ERCC (External RNA Controls Consortium) RNA Spike-In Control Mixes (1:40,000 dilution) (Thermo Fisher Scientific) were added to each of the tubes. The cDNA was amplified with 11 cycles of PCR. For making a library for sequencing, Nextera XT DNA Library Preparation kit (Illumina) was used and 13 cycles of final PCR amplification were done. The obtained library was quality-checked by Bioanalyzer and quantified using Qubit.

### Sequencing data filtering and RNA-seq analysis

Paired-end 50 and 25 bp sequencing was carried out using the Illumina Hiseq2000 system (Illumina). FASTQ files from Illumina sequencing were filtered for low quality reads (<Q20), and low quality bases were trimmed from the ends of the reads (<Q20). Adapters were removed from raw Illumina reads using trim_galore (https://www.bioinformatics.babraham.ac.uk/projects/trim_galore/). Reads were then mapped to the mouse reference genome (mm10) using STAR (Dobin et al., 2013). For the uniquely mapped reads, featureCounts counted and calculated the expression of each gene using fragments per kilo-base of transcript per million mapped (FPKM) fragments value (Liao et al., 2014). Additionally, to remove and identify reads mapped to repeat sequences we used dataset from the UCSC Table browser. Differential expression analysis was performed using the DESeq2 (Love et al., 2014). Heat map and cluster analysis were conducted by the TCC (cluster analysis: based on the unweighted Pair Group Method with Arithmetic mean). Hypergeometric tests were performed to compare the common genes between downregulated DEGs in PIASy-WT-overexpressed embryos and the major ZGA gene list or between downregulated DEGs in PIASy-WT-overexpressed embryos and the two-cell transient gene list; the major ZGA gene and two-cell transient gene lists were obtained from DBTMEE. RNA-seq read alignments were visualized by the Integrative Genomics Viewer (IGV, version 2.3.97; Broad Institute).

### Statistical analysis

For statistical analysis, we used StatView version 5.0 (SAS Institute) and Microsoft Excel, and performed analysis of variance (ANOVA) with an α level of 0.05 to determine statistically significant differences between the means of groups. The number of biological replicates is shown as *n*. We tested the difference in the developmental rate between the experimental and control group using an unpaired *t*-test. For the comparison of the expression profile of Piasy mRNA and protein, the expression level of MERVL mRNA, the developmental rate, and the signal intensity among three experimental groups, the means were compared with a Tukey–Kramer test. Box plot graphs were generated using the BoxPlotR tool (http://shiny.chemgrid.org/boxplotr/).

## Supplementary Material

Supplementary information
