# Supplementary material for: Perturbation of maternal PIASy abundance disrupts zygotic genome activation and embryonic development via SUMOylation pathway
Source: Biol Open. 2019 Oct 22;8(10):bio048652. doi: 10.1242/bio.048652 (PMC6826278; doi:10.1242/bio.048652)
Supplement: Supplementary information [file biolopen-8-048652-s1.pdf]

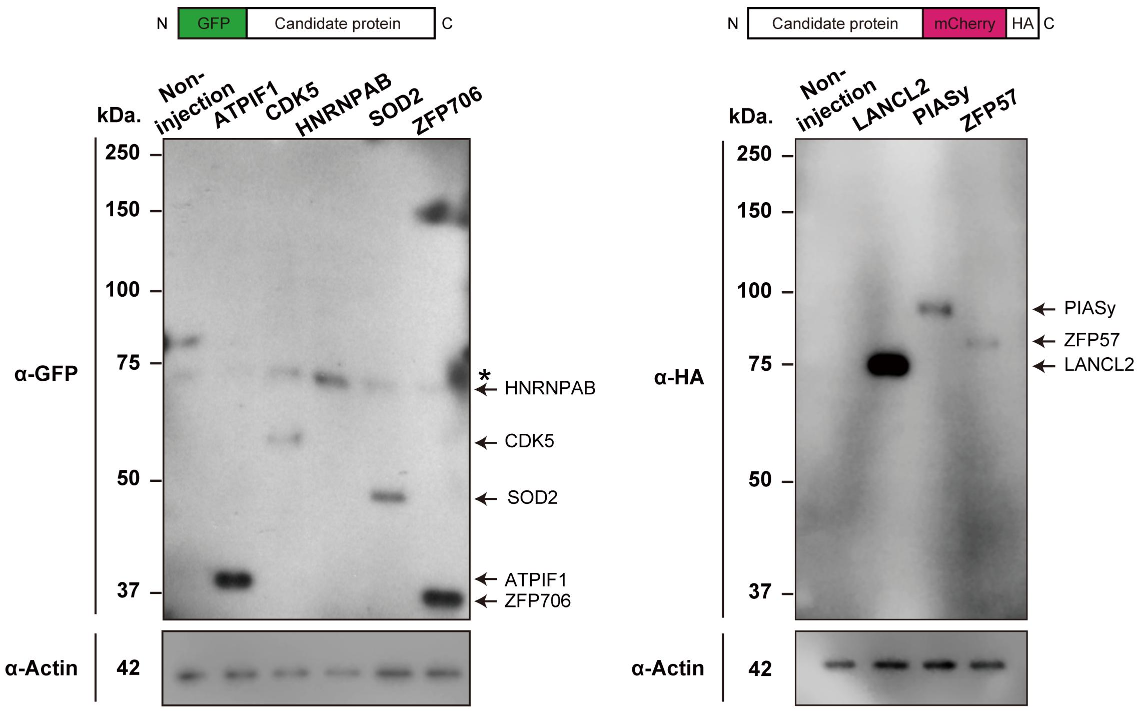

Figure S1. Exogenous expression of candidate proteins by mRNA injection in mouse embryos. Related to Figure 1.

Overexpression of GFP- or mCherry-HA-tagged candidate proteins was detected by Western blotting using anti-GFP antibody (left panel) or anti-HA antibody (right panel). Total proteins from 15 embryos were loaded in each lane. Actin was used as a loading control. Molecular masses (kDa) are shown on the left. * Asterisk indicates a non-specific band.

Biology Open • Supplementary information

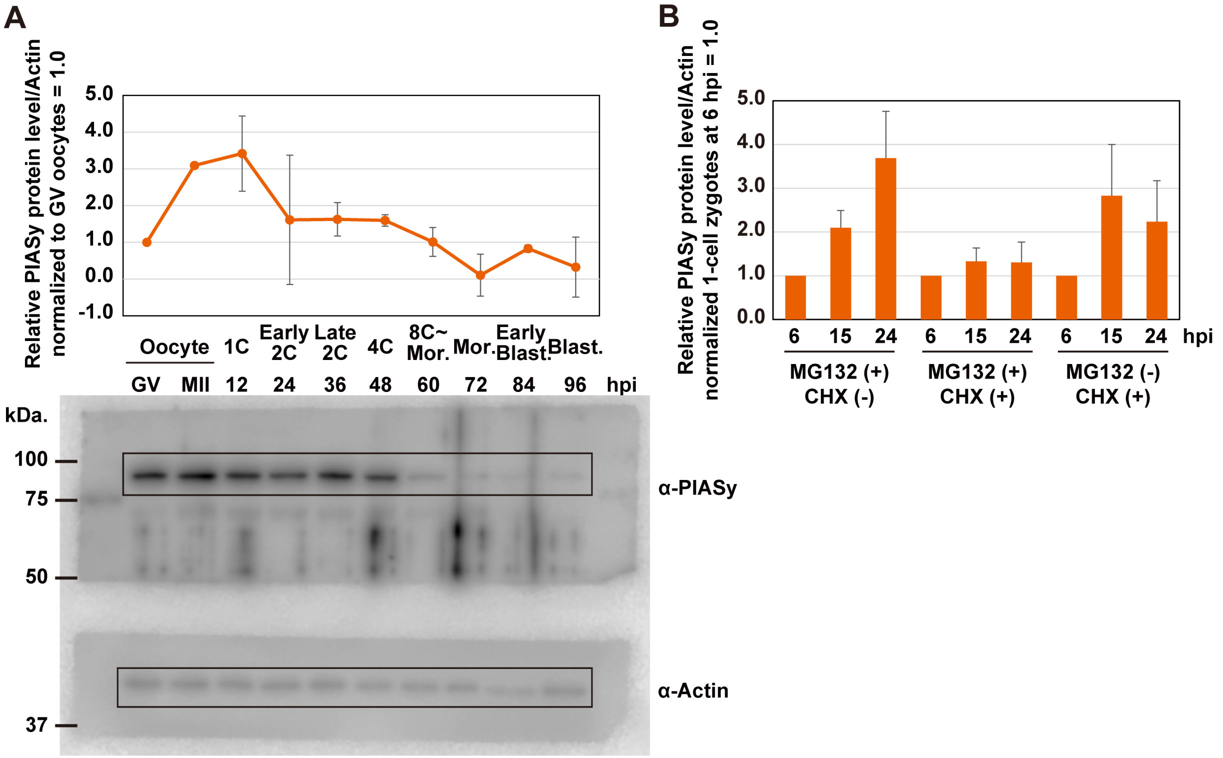

Figure S2. PIASy is expressed in early mouse embryos and degraded by proteasome. Related to Figure 2.

(A) Densitometric quantification analysis of immunoblot bands of endogenous PIASy in Figure 2B. Data represent mean±s.e.m. The lower panel shows uncropped images for Figure 2B. (B) Densitometric quantification analysis of immunoblot bands of PIASy in Figure 2E. Mean+s.e.m is shown.

Biology Open • Supplementary information

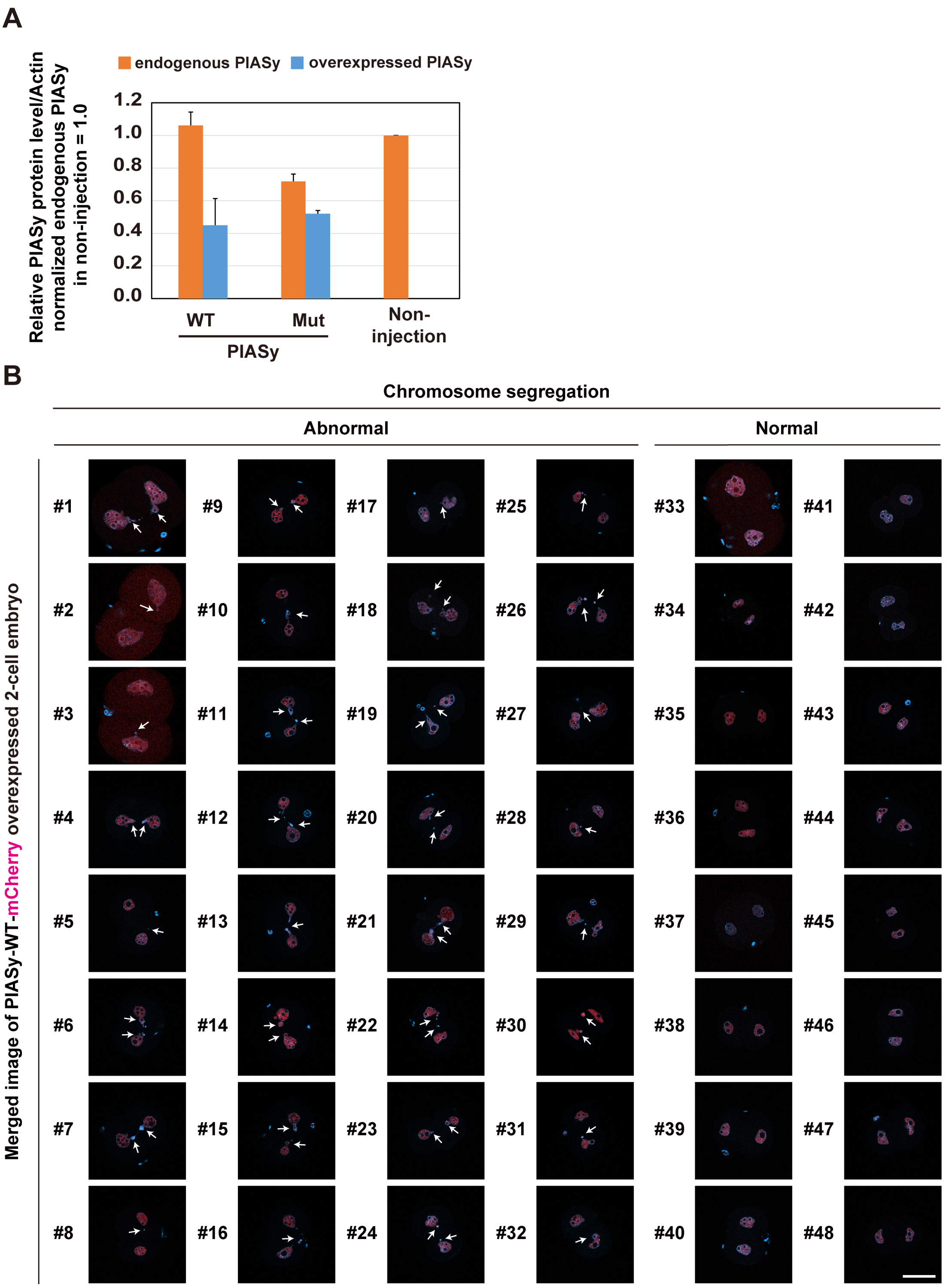

Figure S3. The effect of PIASy overexpression on chromosome segregation. Related to Figure 3.

(A) Densitometric quantification analysis of immunoblot bands of PIASy in Figure 3C. Mean+s.e.m is shown. The data were acquired in duplicates for each sample. Endogenous PIASy (80 kDa; orange bars), overexpressed PIASy (100 kDa; blue bars). (B) Shown are merged images of mCherry (red) and DAPI (blue) in PIASy-WT-overexpressed 2-cell embryo (n=48). Many of embryos indicate abnormal chromosome

segregation such as micronuclei and lagging chromosome. Embryos with abnormal chromosome segregation are labelled as Abnormal, while normal embryos are as Normal. Scale bar: 50 µm. Arrows indicate DNA fragments.

Biology Open • Supplementary information

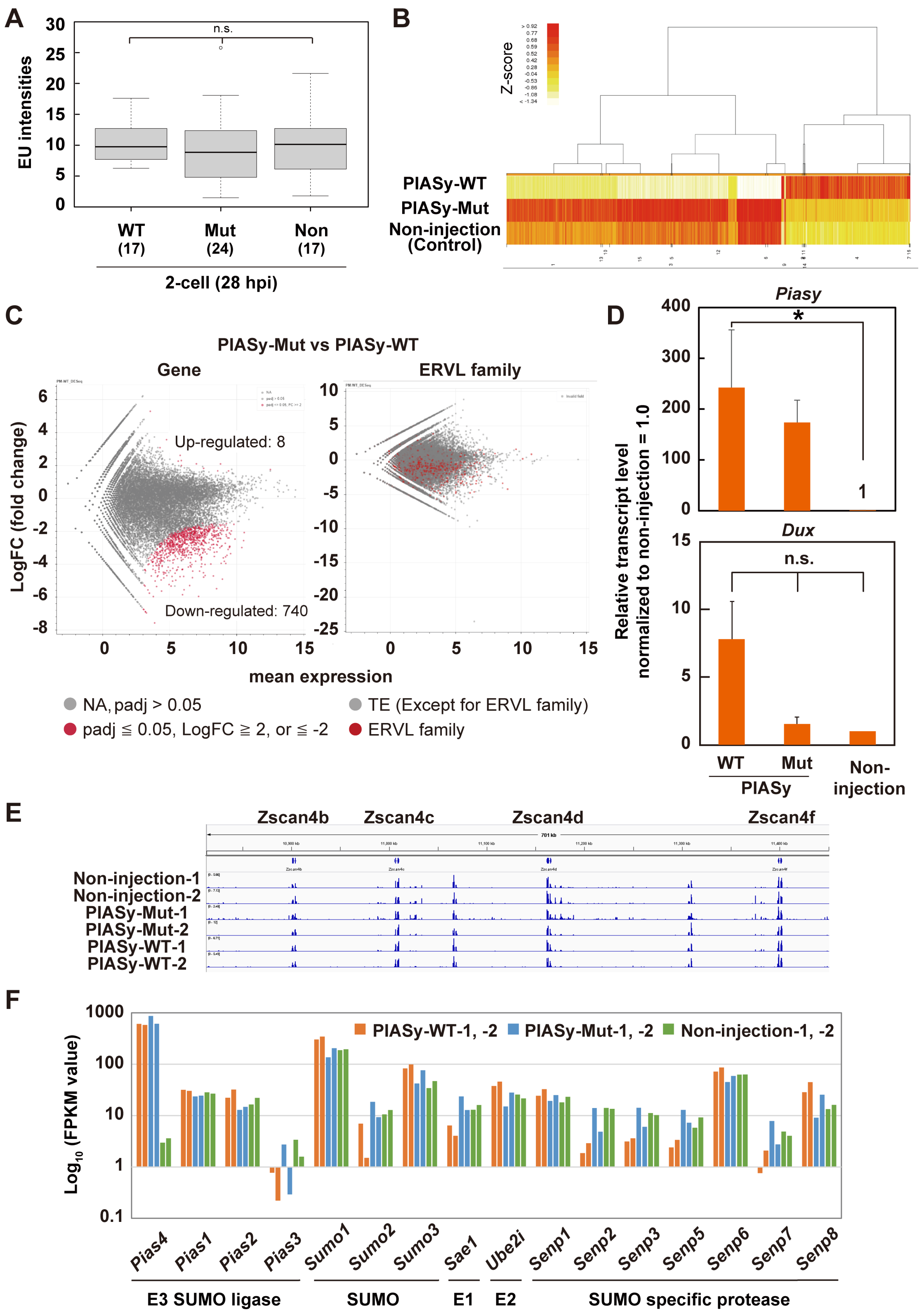

Figure S4. Effects of overexpressing PIASy on zygotic genome activation and SUMO related genes. 'Related to Figure 4.

(A) Quantification of EU intensities in nuclei of examined embryos at 28 hpi (more than 17 embryos were

tested). Significant differences were analyzed with Tukey-Kramer tests. n.s., non-significant. (B) Heat map showing hierarchical clustering for genes with padj <= 0.05 in at least one pair-wise comparison. (C) MA plot displaying differentially expressed genes (left panel) and ERVL family retrotransposons (right panel) in PIASy-Mut versus PIASy-WT embryos. DE genes with adjusted padj <= 0.05 and log FC >= 2, or <= -2 are highlighted in red. padj > 0.05 are highlighted in gray. ERVL family retrotransposons are highlighted in red, while the other retrotransposons are in gray. (D) The upper graph shows *Piasy* mRNA levels in PIASy-WT-overexpressed, PIASy-Mut-overexpressed, and non-injected control 2-cell embryos at 28 hpi using the same samples as Figure 4G. The lower graph shows the *Dux* mRNA levels in PIASy-WT-overexpressed, PIASy-Mut-overexpressed, and non-injected 2-cell embryos at 28 hpi. The expression levels of non-injected embryos were defined as 1. Significant differences were analyzed with Tukey-Kramer tests (* $P < 0.05$). Data represent mean±s.e.m. n.s., non-significant. (E) Track images of RNA-seq reads at the *Zscan4* cluster in PIASy-WT-overexpressed, PIASy-Mut-overexpressed, and non-injected embryos. (F) The expression levels of SUMO-related transcripts in PIASy-WT-overexpressed, PIASy-Mut-overexpressed, and non-injected embryos. The graph shows $\log_{10}$ (FPKM value) of PIASy-WT-1, -2 (orange), PIASy-Mut-1, -2 (blue), and non-injection (green).

Biology Open • Supplementary information

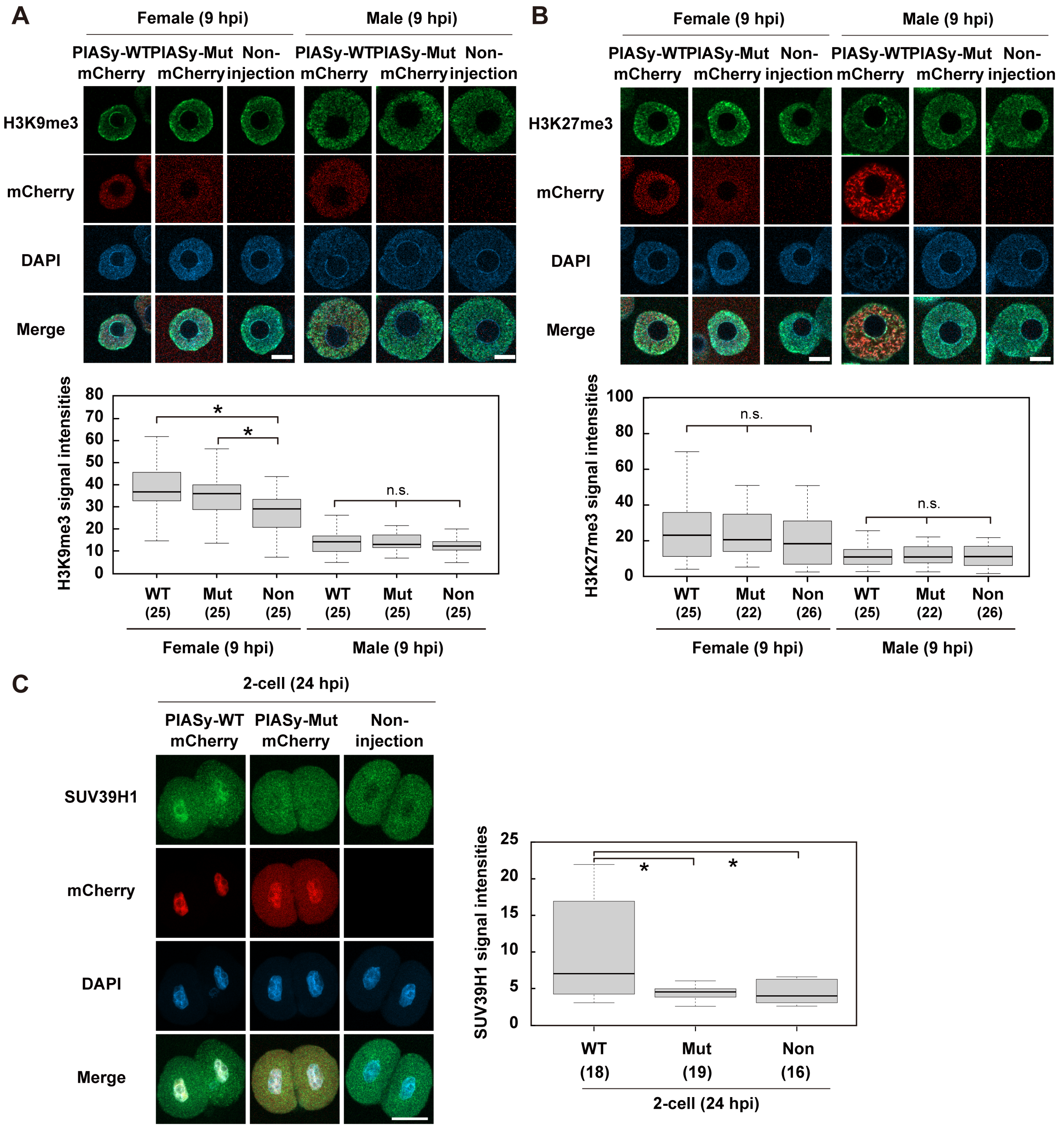

Figure S5. PIASy-WT overexpression causes increased H3K9me3. Related to Figure 5.

(A, B) Immunofluorescence analysis of endogenous H3K9me3 (A) and H3K27me3 (B) in 1-cell zygotes at 9 hpi after overexpression of PIASy-WT or PIASy-Mut. Shown are representative images of female or male pronuclei stained with anti-H3K9me3 antibody or anti-H3K27me3 antibody (green). Non-injected embryos were used as control. All nuclei were stained with DAPI (blue). Scale bars: 10 µm. Lower panels show the quantification of H3K9me3 and H3K27me3 intensities in pronuclei of examined zygotes at 9 hpi (more than 22 zygotes were tested). Significant differences were analyzed with Tukey-Kramer tests (* $P < 0.05$). n.s., non-significant. (C) Subcellular localization of endogenous SUV39H1 in 2-cell embryos at 24 hpi,

overexpressed with PIASy-WT or PIASy-Mut. Shown are representative images of embryos stained with anti-SUV39H1 antibody (green). All nuclei were stained with DAPI (blue). Scale bars: 50 µm. The right panel shows the quantification of SUV39H1 intensities in nuclei of examined embryos at 24 hpi (more than 16 embryos were tested). Significant differences were analyzed with Tukey-Kramer tests (* $P < 0.05$).

Biology Open • Supplementary information

Supplementary Tables

Table S1. 490 candidate proteins (bold and orange characters represent candidate proteins used in this study).

The list of candidate maternal proteins, which are degraded by the UPS during MZT and can contribute to zygotic transcription and normal development. The criteria for selecting the listed proteins are described in the relevant part in the manuscript.

Click here to Download Table S1

Biology Open • Supplementary information

Table S2. Upregulated (non-injection vs PIASy-WT: 339, PIASy-Mut vs PIASy-WT: 13) or downregulated genes (non-injection vs PIASy-WT: 734, PIASy-Mut vs PIASy-WT: 868) in PIASy-WT-overexpressed emrbyos were categorized based on the cluster genes in DBTMEE.

Cluster	Non-injection vs PIASy-WT				PIASy-Mut vs PIASy-WT			
	Up (padj<=0.05, logFC>=1)		Down (padj<=0.05, logFC<=-1)		Up (padj<=0.05, logFC>=1)		Down (padj<=0.05, logFC<=-1)	
	Number of genes	% of genes	Number of genes	% of genes	Number of genes	% of genes	Number of genes	% of genes
maternal RNA	49	47.1	11	4.2	1	25.0	20	6.6
minor ZGA	54	51.9	8	3.0	3	75.0	7	2.3
1C transient	1	0.9	2	0.8	0	0.0	1	0.3
major ZGA	0	0.0	117	44.2	0	0.0	127	41.6
2C transient	0	0.0	43	16.2	0	0.0	49	16.1
MGA	0	0.0	84	31.7	0	0.0	101	33.1
Total	104		265		4		305	

Biology Open • Supplementary information

Table S3. Plasmid and RT-qPCR primers used in this study.

Gene	Forward (5' to 3')	Reverse (5' to 3')	Application
Atpif1	CACCGCAGGCTCGGCGTTGGCAGTTCG	TCAATGATTATTCTTTAGTTG	Cloning
Cdk5	CACCCAGAAATACGAGAAACTGGAG	CTATGGGGGACAGAAGTCAG	Cloning
Hnrnpab	CACCTCGGACGCGGCTGAGGAGCAGC	TCAGTATGGCTTGTAGTTATTCTG	Cloning
Lancl2	CACCATGGGCGAGACCATGTCAAAGAG	ATCCTTCTGCAAAAAGCCAAGTTC	Cloning
Piasy	CACCATGGCGGCAGAGCTGGTGGAGG	GCACGCGGGCACCAGGCCTTTC	Cloning
Sod2	CACCTTGTGTCGGGCGGCGTGC	TCACTTCTTGCAAGCTGTG	Cloning
Zfp57	CACCATGGCAGCTAGGAAACAGTCTTCC	GTCCGAATCTTCTTCTGTCACAGG	Cloning
Zfp706	CACCGCTCGTGGACAGCAGAAGATTC	TTATGCCTGAACATCAGC	Cloning
Piasy-C335F (Mut)	CGTGCAGAGACCTTCGCACACCTGC	GCAGGTGTGCGAAGGTCTCTGCACG	Mutagenesis
Piasy	TCGCTGTGAAGGTCAACCAC	AGGGATAGTAGCCCGGCACT	RT-qPCR
MERVL	TGCTGCGTGAGACTGAGTAATTGG	TCGCAGCTGTGAATGGAAGT	RT-qPCR
Dux	CATGCCTCAAAGAGGTCCATC	TCTCAAAGGCTTGCCCTAGGA	RT-qPCR

Biology Open • Supplementary information

Table S4. Antibodies used in this study.

Antibody name	Company	Catalog No.	Working dilution WB	IF
anti-PIASy antibody	Sigma-aldrich	SAB4502145	1:1,000	1:500
anti-HA antibody	Sigma-aldrich	H9658	1:2,500	
anti-GFP (9F9.F9) antibody	Novus Biologicals	NB600-597	1:3,000	
anti-Actin antibody	Sigma-aldrich	A5441	1:20,000	
anti-Ub (FK2) antibody	Enzo Life Sciences	BML-PW8810	1:1,000	
anti-SUMO-1 (21C7) antibody	Thermo Fisher	33-2400		1:500
anti-SUMO-2/3 (8A2) antibody	abcam	ab81371		1:2,000
anti-H3K9me3 antibody	abcam	ab8898		1:5,000
anti-H3K27me3 antibody	active motif	39157		1:1,500
anti-SUV39H1 (D11B6) antibody	Cell Signaling	8729		1:500
Donkey anti-rabbit IgG horseradish peroxidase (HRP) conjugate	Millipore	AP182P	1:50,000	
Donkey anti-moue IgG HRP conjugate	Millipore	AP192P	1:50,000	
Alexa Fluor 488-labeled goat anti-mouse IgG antibody	Invitrogen	A-11001		1:2,000
Alexa Fluor 488-labeled goat anti-rabbit IgG antibody	Invitrogen	A-11008		1:2,000

Biology Open • Supplementary information
